# Adenosquamous Carcinoma of the Lung: Survival, Radiologic Findings, PD-L1, and Driver Mutations

**DOI:** 10.3390/jcm13195711

**Published:** 2024-09-25

**Authors:** Oliver Illini, Hannah Fabikan, Eva Fischer, Anna Sophie Lang-Stöberl, Dagmar Krenbek, Christa Jarius, Shokoufa Azarnia-Medan, Stefan Gasser, Maximilian Johannes Hochmair, Christoph Weinlinger, Arschang Valipour, Stefan Watzka

**Affiliations:** 1 Department of Respiratory and Critical Care Medicine, Clinic Floridsdorf, Vienna Healthcare Group, Bruenner Straße 68, 1210 Vienna, Austria; 2 Karl Landsteiner Institute of Lung Research and Pulmonary Oncology, Clinic Floridsdorf, Bruenner Straße 68, 1210 Vienna, Austria; 3 Department of Anesthesiology and Intensive Care, Clinic Donaustadt, Vienna Healthcare Group, Landobardenstraße 122, 1220 Vienna, Austria; 4 Department of Pathology, Clinic Floridsdorf, Vienna Healthcare Group, Bruenner Straße 68, 1210 Vienna, Austria; 5 Diagnostic Center Floridsdorf, Mitterhofergasse 2/8, 1210 Vienna, Austria; 6 Unit for Interventional Radiology, Institute for Diagnostic and Interventional Radiology, KABEG Klinikum Klagenfurt am Wörthersee, Feschnigstraße 11, 9020 Klagenfurt, Austria; 7 Department of Thoracic Surgery, Clinic Floridsdorf, Vienna Healthcare Group, Bruenner Straße 68, 1210 Vienna, Austria; stefan.watzka@gesundheitsverbund.at; 8 Karl Landsteiner Institute for Clinical and Translational Thoracic Surgical Research, Clinic Floridsdorf, Bruenner Straße 68, 1210 Vienna, Austria

**Keywords:** adenosquamous carcinoma, lung cancer, EGFR, PD-L1, pathological subtypes, prognostic factors

## Abstract

**Background:** Adenosquamous carcinoma of the lung (ASC) is a rare non-small-cell lung cancer (NSCLC) subtype combining components of squamous cell carcinoma (SCC) and adenocarcinoma (AC). Data on ASC, particularly in Caucasian populations, are limited. **Methods:** We reviewed clinicopathological and radiological characteristics of ASC patients diagnosed between 1996 and 2023. Patients were classified into AC-predominant ASC (AC-ASC) and SCC-predominant ASC (SCC-ASC) groups for analysis. **Results:** Among the 66 patients included, the median overall survival was 41.7 (95% CI, 25.0–54.4), while it was 48.1 (95% CI, 27.3–88.0) in patients treated with curative surgery (n = 44) and 15.3 (95% CI, 6.5–42.6) months for palliative patients (n = 22). The five-year survival rates were 39% and 26%, respectively. Recurrence occurred in 43% of stage I patients and was associated with worse survival (HR 3.303 (95% CI, 1.10–9.89) *p* = 0.033). AC-ASCs (n = 17) more frequently showed air-bronchogram (*p* = 0.002) and pleural effusions (*p* = 0.054) compared to SCC-ASCs (n = 26). SCC-ASCs exhibited more vascular invasion (*p* = 0.006) and PD-L1 values between 1 and 49% (TPS) (*p* = 0.032). The subtype did not influence survival. EGFR and ALK alterations were found in 17% and 2% of patients, respectively. **Conclusions:** Despite early-stage disease, ASC patients had a high recurrence rate, associated with worse survival. Clinicopathologic differences between AC-ASCs and SCC-ASCs did not influence survival.

## 1. Introduction

Adenosquamous carcinoma of the lung (ASC) is a histologic subtype of non-small cell lung cancer (NSCLC) that combines components of both squamous cell carcinomas (SCC) and adenocarcinomas (AC). With a prevalence of 0.4 to 4% of all NSCLC, ASC is very rare [1]. Defined by the World Health Organization (WHO) pathological classification system in 2015, ASC has been categorized as a carcinoma with both AC and SCC components accounting for at least 10% of the tumor [2]. 

However, it is important to note that ASC is more than merely a combination of two histologic subtypes, as it presents with unique features, and seems to be more aggressive and associated with a worse prognosis compared to ACs and SCCs [1]. 

Due to its low incidence, the ASCs’ clinicopathological and prognostic features are yet incompletely understood [3]. Moreover, most studies focusing on ASC are based on Asian cohorts and only a few studies have analyzed the ASC in the Caucasian population [4,5,6]. To improve our understanding of pathologic and radiographic features and prognostic predictions of ASC of the lung in the Caucasian population, we analyzed real-world data of all consecutive ASC patients treated at our clinic.

## 2. Patients and Methods

### 2.1. Study Design

This was a retrospective single-center analysis of patients with ASC, who were consecutively treated at the Department of Thoracic Surgery and the Department of Respiratory and Critical Care Medicine at the Clinic Floridsdorf, Vienna, Austria. The aim of this study was to describe and analyze patient and tumor characteristics including imaging and histopathological studies, with special attention on structural components and genetic mutations, as well as treatment strategies and their impact on overall survival in a real-world setting. 

This study was reviewed and approved by the ethics committee of the city of Vienna, Austria (EK-14-030-VK; approval date: 10 April 2014; amendment 18 August 2016) and conducted in accordance with the Declaration of Helsinki [7]. Due to the retrospective design of this study, informed consent from each patient was waived.

### 2.2. Patients

Patients were over the age of 18 with a histological-confirmed ASC from our database between January 1996 and March 2023. Tumor staging was based on the 8th edition lung cancer stage classification [8] 

All histopathological specimens were independently reviewed by two clinical pathologists (CJ and DK). The ASC subtype was confirmed by histomorphological features: adenomatous patterns, acinar, papillary, micropapillary, lepidic and/or mucin for AC component, and intercellular bridges keratinization for SC component; in poorly differentiated solid areas, immunohistochemical stains (IHC) p40 and TTF-1 were conducted to distinguish between the components of AC and SCC. Patients were categorized according to the predominance of ≥60% of the histological components as either AC-predominant (AC-ASC), SCC-predominant (SCC-ASC), or as structurally balanced (BAL-ASC) with both components accounting for 40–59% of the tumor [9].

All patients’ samples were assessed for epidermal growth factor receptor (EGFR), ROS protooncogene 1 (ROS-1), anaplastic lymphoma kinase (ALK) mutations, and programmed death-ligand 1 (PD-L1) status. Further information on the assessment procedure can be found in the Supplementary Materials. All CT images were independently examined by two radiologists specialized in thoracic radiology (SG, SA). Special consideration was given to the characterization of the tumor itself regarding the size, location, and shape of the margins. Furthermore, internal characteristics and patterns were analyzed regarding air bronchogram, pleural tags, the existence of caverns or vesicles, and the existence of inflammatory changes surrounding the tumor. Additionally, the presence of involved lymph nodes, pericardial effusion, and pleura effusion was evaluated.

### 2.3. Statistical Analysis

Patient and tumor characteristics are reported descriptively and shown as a mean with standard deviation (SD) or median with quartiles. Categorical data are shown as frequencies and proportions. Differences between groups were assessed either using the student t-test for normally distributed variables of the Kruskal–Wallis test in case of no normal distribution. Categorical variables were compared using contingency table analysis and the χ^2^ test. Overall survival (OS) was defined as the time interval from the date of diagnosis to the date of death or censoring date. Patients alive or lost to follow-up were censored with the date of the last contact. 

The median OS was calculated using the Kaplan–Meier estimator and a confidence interval (CI) of 95%. The median follow-up was calculated using the reversed Kaplan–Meier estimator. Univariate potential prognostic predictors for survival such as age, sex, smoking status, tumor stage, radiological characteristics, predominant histological components, and genetic mutations were assessed using the log-rank test with a level of significance of 5% (chi-square *p* = 0.05), followed by stepwise forward multivariate Cox regression analysis. A two-sided *p*-value < 0.05 was considered statistically significant. All data were analyzed with Stata version 14 (StataCorp LP, College Station, TX, USA).

## 3. Results

### 3.1. Patient Characteristics 

**Figure 1:**
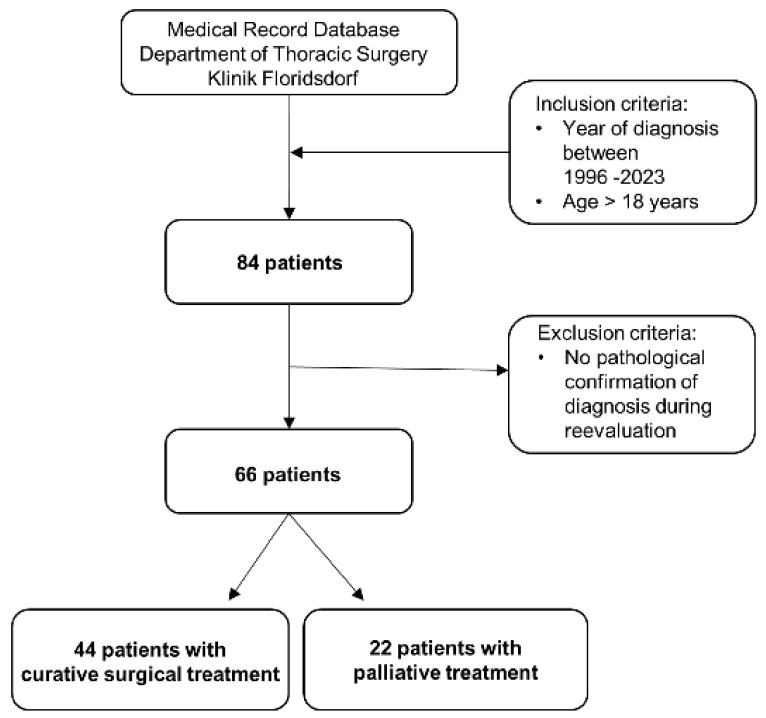
Flowchart of the study.

Overall, 84 patients were diagnosed with ASC. Of these patients, 18 had to be excluded after revision of biopsy specimens and histological samples (Figure 1). Hence, 66 patients were included in the study. The baseline characteristics are reported for all patients and according to treatment intent in Table 1. Due to the retrospective nature of the study, some characteristics (e.g., smoking status and pack years) were not documented for all of the patients included. An activating EGFR mutation was found in 11 patients (17%), consisting of 5 patients with an exon 19 deletion (Del19), 4 patients with an exon 21 Leu858Arg substitution (L858R), and 1 patient with both a G719X and exon 20 insertion (ins20). An ALK fusion was found in 1 patient (2%). No ROS-1-fusion was found. Eleven patients (17%) showed a PD-L1 status of 50% or higher. Retrospective radiological characterization was possible in 50 cases (Table 1).

Surgery was performed by thoracotomy (77%) or video-assisted thoracoscopic surgery (23%). Resection margins of R0 were achieved in 42 patients (95%). Most of those patients were diagnosed with an SCC-ASC (59%), followed by patients with AC-ASC (39%). Table 2 shows all of the pathological findings in patients treated with curative surgery. Table 3 shows the perioperative treatment characteristics. For an assessment of tumor markers, see Supplementary Table S1. 

The radiological findings showed differences between patients with an AC-ASC and an SCC-ASC regarding the air-bronchogram (50% vs. 5%, *p* = 0.002) and pleural effusion (17% vs. 0%, *p* = 0.054). In pathologic analysis, the vascular invasion was more often found in SCC-ASCs than in AC-ASCs (35% vs. 0%, *p* = 0.006) and we found differences in PD-L1 expression (*p* = 0.032, see Supplementary Table S2). There were no differences regarding stage, size, mutations, or invasion of the pleura viscera.

### 3.2. Recurrence 

Recurrence (median follow-up of 180 months) occurred in 43% of curative-treated patients. In detail, 43% of patients with stage I disease at initial diagnosis, 57% with stage II, and 39% with stage III had a recurrence. The median progression-free survival (PFS) in surgically treated patients was 23 (95% CI: 14.6–64.7) months. Recurrence occurred in 29% of patients diagnosed with an AC-ASC and 50% of patients with an SCC-ASC (*p* = 0.181). There was no significant association of any histologic or radiologic finding and recurrence.

### 3.3. Survival

The median follow-up time was 180 (95% CI: 67.2–246.8) months. The median OS of all patients was 41.7 (95% CI: 25.0–54.4) months and the 5-year survival rate (5-year OS) was 39%. The five-year OS rate in stage I was 63%, in stage II was 50%, in stage III was 42%, and in stage IV was 6% (*p* = 0.009).

After a median follow-up time of 180 (57.5; NA) months, the median OS of patients with curative surgery was 48.1 (95% CI: 27.3–88.0) months and the 5-year OS was 48%. Palliative-treated patients had an inferior median OS of 15.3 (95% CI: 6.5–42.6) months (*p* = 0.077) and a 5-year OS of 23%.

The associations of prognostic parameters with OS are shown in Table 4. The 5-year OS of curatively treated patients with high and low serum CEA levels was 33% and 64% (*p* = 0.147), and the OS rate at the end of the study was 11% and 36% (*p* = 0.190), respectively. The 5-year OS with AC-predominance was 59% compared to 42% in patients with SCC-predominance (*p* = 0.357) with a median OS of 45.8 (95% CI: 26.0–88.0) and 54.4 (95% CI: 24.6–112.6) months, respectively (*p* = 0.471). Elderly patients showed a significantly longer median OS of 64.7 (95% CI: 32.4–140.4) months compared to patients under 65 years with 24.8 (95% CI: 12.3–41.7) months (*p* = 0.013). However, 80% of those younger patients were diagnosed at stage III and only 7% at stage I. In contrast, patients ≥65 years were diagnosed at stage III in 38% and 45% at stage I. Patients with recurrence showed a reduced median OS of 32.4 (95% CI: 24.8–54.4) months vs. 88.0 (95% CI: 33.5–213) months (*p* = 0.009). Patients with spiculated tumor contours showed a worse median OS of 45.8 (95% CI: 24.8–55.1) months compared to patients with smooth tumor contours (112.6 (95% CI: 1.1-NA), *p* = 0.037). Additional treatment with chemo- and/or immunotherapy showed a numerically prolonged survival with 111.2 (95% CI: 13.4–150.3) months vs. 45.8 (95% CI: 24.9–64.7) months without neoadjuvant or adjuvant treatment (*p* = 0.325). Those patients with chemo- and/or immunotherapy were more often diagnosed at stage III (63% vs. 46%, *p* = 0.372).

**Figure 2:**
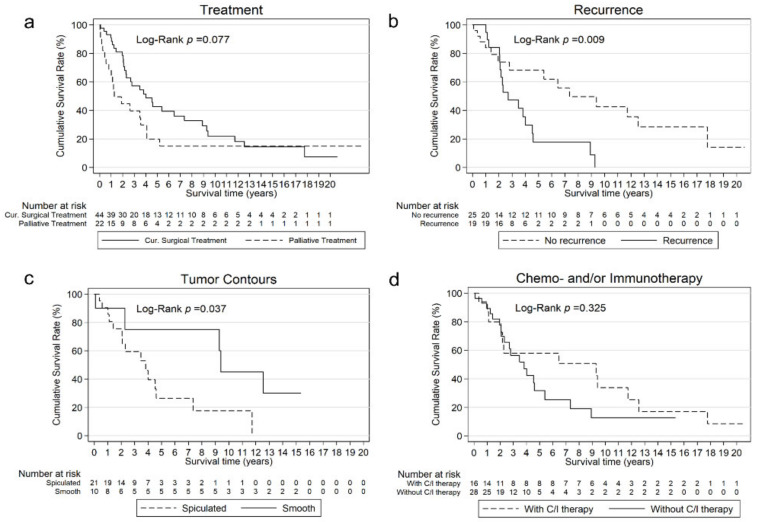
Overall survival (OS) analysis. (**a**). OS curves for all patients with curative surgical or palliative treatment (*p* = 0.077). (**b**). OS curves for patients with curative surgical treatment who had a recurrence and who did not have a recurrence (*p* = 0.009); (**c**). OS curves for patients with curative surgical treatment whose tumor contours were either spiculated or smooth (*p* = 0.037); (**d**). OS curves for patients with curative surgical treatment with and without additional chemo- and/or immunotherapy (*p* = 0.325).

Multivariate analysis revealed recurrence to be an independent prognostic factor of survival (*p* = 0.033). Patients with recurrence had a significantly higher mortality rate compared to patients without recurrence (HR 3.303 (95% CI: 1.10–9.89)). The cumulative survival curves according to treatment intent and of patients with surgery with significant prognostic variables are shown in Figure 2.

## 4. Discussion

This study provides comprehensive long-term real-world data of 66 Caucasian patients diagnosed with ASC with a follow-up time of more than 15 years. We describe and assess prognostic implications of clinical, radiological, and histological characteristics.

ASC has been associated with poor prognosis [1]. Numerous studies show worse outcomes for ASC than for any other type of NSCLC, reporting a 5-year OS from 6.2% to 37% [4,10,11,12]. In comparison, we report a rather high 5-year survival of 39%, resembling those reported in AC or SCC from ASC comparison cohorts [10,12]. Nevertheless, we observed high recurrence rates in early-stage patients. Compared to Handa et al., who reported a rate of 33%, we found an even higher recurrence rate of 43% in stage I disease. These data contrast substantially lower recurrence rates of 5% and 17% in a similar cohort of stage I AC and SCC patients, respectively [3]. In our study, the 5-year OS in stage I was 63%, which was similar to the 67% reported by Handa et al. [3]. Recurrence was the only independent predictor of survival in our study (HR 3.303 (95% CI: 1.10–9.89) *p* = 0.033). As risk factors for recurrence, especially in early-stage ASC, high serum CEA and vascular invasion have been reported [3]. We observed a higher median survival of 106.8 months and a 5-year OS of 64% in surgically treated patients with low CEA compared to 33.5 months and 33% in those with higher serum levels. These results are in accordance with published data on the association of preoperative CEA with survival [13]. Furthermore, EGFR mutation has been previously reported to be another prognostic factor of ASC. In a European study with only 14 patients, EGFR mutations were detected in 29% of ASCs, which even exceeds the mutation rates of adenocarcinomas [5,14]. In our study, one-fifth of patients presented with an oncologic driver mutation (tested for EGFR, ALK, and ROS-1), indicating the importance of reflex testing in all patients with NSCLC including ASC. With the advent of selective tyrosine kinase inhibitors, the treatment options and prognosis for patients with EGFR- and ALK-positive NSCLC have significantly improved [15,16,17,18,19]. Some of those studies have also included patients with ASC and the efficacy of targeted therapy in ASC seems to be comparable to that in adenocarcinoma [20]. EGFR mutations were found in 17% of our patients, which is similar to rates seen in ACs in Europe [14,21]. While EGFR mutations have been correlated with a significantly better 3-year survival [22], we found no longer median survival for EGFR-positive ASC patients. Regarding perioperative treatment, we observed a numerically higher survival in patients treated with chemo- and/or immunotherapy in addition to surgical removal of the tumor (111.2 vs. 45.8 months) even though those patients were more often diagnosed in the advanced tumor stage. This is especially noteworthy as treatment with chemotherapy alone has been reported to not improve the prognosis of ASC [23].

Regarding radiologic characteristics, smooth tumor contours were linked to better prognosis than spiculated contours in our study (median OS 112.6 vs. 45.8 months). NSCLC with irregular contours are more likely to be invasive according to Yip et al. [24] However, our multivariate analysis could not confirm contours as an independent prognostic factor. The apportionment of glandular and squamous components of the ASC has been argued to determine prognosis [25]. In accordance with several other reports, we found no differences in prognosis according to the predominant components [4,10,26].

Immune checkpoint inhibitors targeting PD1/PD-L1, have revolutionized the treatment of lung cancer over the last decade [27]. In our analysis, SCC-ASCs presented more often with PD-L1 values between 1 and 49% and vascular invasion. Higher rates of PD-L1 in SCC-ASCs than in AC-ASCs and similar expression levels in SCC-ASCs and SCC as well as in AC-ASCs and AC have been reported [28,29]. Therefore, it seems that the PD-L1 expression in ASCs resembles their histological subtype, while it remains uncertain as to whether ASC patients gain the same benefit from immunotherapy regimens [30].

Our study is limited by its retrospective nature and includes only a limited number of patients due to the rarity of the disease, different therapeutics, and treatment regimens. However, the long observation time allowed us to analyze prognostic factors over a long period of time and assess their implications on overall survival.

## 5. Conclusions

In conclusion, our study demonstrates that a survival rate comparable to other NSCLC subtypes can be achieved in ASC patients. Nevertheless, we found that ASCs have a high recurrence rate already at an early stage and that recurrence predicts survival. The additional treatment with chemo- and/or immunotherapy may be able to improve the median survival in curative-treated patients. Moreover, we report high rates of EGFR mutations and a numerically reduced median survival in patients with high serum CEA and spiculated tumor contours. Although we observed different radiologic and histological findings in patients with morphological AC-ASC or SCC-ASC types, they did not influence survival probability. Our findings lay the foundation for further studies needed to improve treatment and prognosis in patients with ASC. 

## Figures and Tables

**Table 1:** Patient characteristics according to treatment.

Characteristics at Diagnosis	All Patients (n = 66)	Patients with Curative Surgical Treatment (n = 44)	Patients with Palliative Treatment (n = 22)
Age, years			
Median (range)	70 (37–92)	71 (37–92)	69 (39–82)
Age groups, n (%)			
<65	23 (35)	15 (34)	8 (36)
≥65	43 (65)	29 (66)	14 (64)
Gender, n (%)			
Male	35 (53)	21 (48)	14 (64)
Female	31 (47)	23 (52)	8 (36)
Ethnicity, n (%)			
Asian	2 (3)	1 (2)	1 (5)
Non-Asian	64 (97)	43 (98)	21 (95)
Smoking status, n (%)	(n = 62)	(n = 41)	(n = 21)
Never smoker	13 (21)	10 (24)	3 (14)
Former smoker	26 (42)	17 (41)	9 (43)
Current smoker	23 (37)	14 (34)	9 (43)
Pack years, n (%)	(n = 36)	(n = 23)	(n = 13)
Smoker (<30 py)	8 (22)	6 (26)	2 (15)
Heavy smoker (≥30 py)	28 (78)	17 (74)	11 (85)
Stage at initial diagnosis, n (%)			
Stage I	16 (24)	14 (32)	2 (9)
Stage Ia	6 (9)	4 (9)	2 (9)
Stage Ib	10 (15)	10 (23)	0 (0)
Stage II	10 (15)	7 (16)	3 (14)
Stage IIa	2 (3)	2 (5)	0 (0)
Stage IIb	8 (12)	5 (11)	3 (14)
Stage III	24 (36)	23 (52)	1 (5)
Stage IIIa	18 (27)	17 (39)	1 (5)
Stage IIIb	6 (9)	6 (14)	0 (0)
Stage IV	16 (24)	0 (0)	16 (73)
Stage IVa	14 (21)	0 (0)	14 (64)
Stage IVb	2 (3)	0 (0)	2 (9)
**Pathological characteristics**	All patients (n = 66)	Patients with curative surgical treatment (n = 44)	Patients with palliative treatment (n = 22)
PD-L1 status, n (%)			
Negative (<1%)	25 (38)	16 (36)	9 (41)
1–49%	30 (45)	21 (48)	9 (41)
50–89%	6 (9)	3 (7)	3 (14)
≥90%	5 (8)	4 (9)	1 (5)
EGFR-mutation ^1^, n (%)			
Positive	11 (17)	7 (16)	4 (18)
Negative	55 (83)	37 (84)	18 (82)
ALK-fusion, n (%)			
Positive	1 (2)	1 (2)	0 (0)
Negative	65 (98)	43 (98)	22 (100)
ROS-1-fusion, n (%)			
Positive	0 (0)	0 (0)	0 (0)
**Radiological characteristics**	All patients (n = 50)	Patients with curative surgical treatment (n = 33)	Patients with palliative treatment (n = 17)
Tumor location, n (%)			
Peripheral	33 (66)	22 (67)	11 (65)
Central	17 (34)	11 (33)	6 (35)
Cavitation, n (%)			
Yes	4 (8)	2 (6)	2 (12)
No	46 (92)	31 (94)	15 (88)
Cysts, n (%)			
Yes	2 (4)	1 (3)	1 (6)
No	48 (96)	32 (97)	16 (94)
Tumor contours, n (%)			
Spiculated	32 (64)	21 (64)	11 (65)
Smooth	15 (30)	10 (30)	5 (29)
Lobulated	3 (6)	2 (6)	1 (6)
Air bronchogram, n (%)			
Yes	9 (18)	7 (21)	2 (12)
No	41 (82)	26 (79)	15 (88)
Inflammatory changes, n (%)			
Yes	15 (30)	10 (30)	5 (29)
No	35 (70)	23 (70)	12 (71)
Pleural or pericardial effusion, n (%)			
Yes	8 (16)	2 (6)	6 (35)
No	42 (84)	31 (94)	11 (65)
Pleura tag, n (%)			
Yes	24 (48)	15 (45)	9 (53)
No	26 (52)	18 (55)	8 (47)

^1^ Overall, 5 patients with an exon 19 deletion (Del19), 4 patients with an exon 21 Leu858Arg substitution (L858R), 1 patient with a G719X mutation, and 1 patient with an exon 20 insertion (ins20). Percentage may not equal to 100 due to rounding.

**Table 2:** Pathological characteristics in patients with curative surgical treatment.

Pathological Characteristics	(n = 44)
**Structural components,** n (%)	
AC-ASC	17 (39)
SCC-ASC	26 (59)
BAL-ASC	1 (2)
**T-stage,** n (%)	
T1	12 (27)
T1a ^1^	2 (5)
T1b	7 (16)
T1c	3 (7)
T2	21 (48)
T2a	14 (32)
T2b	7 (16)
T3	7 (16)
T4	4 (9)
**N-stage,** n (%)	
N0	24 (55)
N1	8 (18)
N2	11 (25)
N3	1 (2)
**Tumor size** (cm)	
Median (Range)	3.5 (0–10.0)
Groups, n (%)	
<5 cm	37 (84)
≥5 cm	7 (16)
**Vascular invasion,** n (%)	
V0	35 (80)
V1	9 (20)
**Invasion of the pleura visceralis,** n (%)	
PL0	31 (70)
PL1	10 (23)
PL2/PL3	3 (7)
**Resection margin,** n (%)	
R0	42 (95)
R1	2 (5)

AC-ASC: Adenocarcinoma-predominant adenosquamous carcinoma, BAL-ASC: structurally balanced adenosquamous carcinoma, SCC-ASC: squamous-cell carcinoma-predominant adenosquamous carcinoma, VATS: video-assisted thoracoscopic surgery; ^1^ one of the patients was diagnosed with T1a(mi); percentage may not equal to 100 due to rounding.

**Table 3:** Perioperative treatment characteristics in patients with curative surgical treatment.

Perioperative Treatment Characteristics	(n = 44)
**Chemotherapy and/or immunotherapy,** n (%)	
Neoadjuvant	5 (11)
Adjuvant	5 (11)
Neoadjuvant and Adjuvant	6 (14)
No chemotherapy	28 (64)
**Treatment with targeted therapy,** n (%) ^1^	
Neoadjuvant	1 (2)
Adjuvant	4 (9)
Neoadjuvant and Adjuvant	1 (2)
No targeted therapy	38 (86)
**Radiation therapy,** n (%)	
Adjuvant	7 (16)
**Type of operation,** n (%)	
(Bi-)Lobectomy	36 (82)
Pneumonectomy	6 (14)
Segmentectomy	2 (5)
**Surgical technique,** n (%)	
Thoracotomy	34 (77)
VATS	10 (23)
**Lymphadenectomy,** n (%)	
Yes	41 (93)
No	3 (7)

^1^ Neoadjuvant therapy was conducted with afatinib; adjuvant therapy was conducted with erlotinib, crizotinib, or gefitinib; the patient receiving neoadjuvant and adjuvant therapy was treated with afatinib in both settings; percentage may not equal to 100 due to rounding.

**Table 4:** Univariate analysis of prognostic parameters in adenosquamous patients with curative surgical treatment.

Variable	N (%)	Median Survival Time (95% CI)	*p*-Value
n = 44			
**Age**, years			
<65	15 (34)	24.8 (12.3; 41.7)	0.013
≥65	29 (66)	64.7 (32.4; 140.4)	
**Gender**			0.766
Male	21 (48)	45.8 (23.4; 77.4)	
Female	23 (52)	55.1 (24.8; 112.6)	
**Smoking status**		27.7 (12.3; NA) 64.7 (32.4; 112.6)	0.708
Never smoker	10 (23)		
Smoking history	31 (70)		
Unknown (excluded)	3 (7)		
**Structural components**			0.471
AC-ASC	17 (39)	45.8 (26.0; 88.0)	
SCC-ASC	26 (59)	54.4 (24.6; 112.6)	
BAL-ASC (excluded)	1 (2)		
**TNM-stage**			0.715
Stage I	14 (32)	55.1 (27.7; 77.4)	
Stage II	7 (16)	45.8 (4.5; NA)	
Stage III	23 (52)	32.4 (14.9; 112.6)	
**Lymph nodes**			0.459
**N0**	24 (55)	55.1 (27.2; 88.0)	
**N1**	8 (18)	106.9 (24.6; NA)	
**N2**	11 (25)	17.0 (11.8; 33.5)	
**N3**	1 (2)		
**Size of tumor**			0.876
<5 cm	37 (84)	54.4 (27.3; 106.9)	
≥5 cm	7 (16)	32.4 (7.1; NA)	
**Vascular invasion**			0.428
V0	35 (80)	41.7 (24.8; 88)	
V1	9 (20)	55.1 (27.7; NA)	
**Visceral pleural invasion**			0.740
PL0	31 (70)	64.7 (25.0; 111.2)	
PL1	10 (23)	45.8 (12.3; NA)	
PL2/PL3	3 (7)	27.3 (4.5; NA)	
**PD-L1 status**			0.884
Negative	16 (36)	33.5 (13.4; 213.0)	
1–49%	21 (48)	48.1 (24.6; 106.9)	
≥50%	7 (16)	111.2 (26.0; NA)	
**Target mutation (EGFR, ALK)**			0.636
Positive	8 (18)	41.7 (11.8; NA)	
Negative	36 (82)	54.4 (27.0; 88.0)	
**EGFR status**			0.416
Positive	7 (16)	41.7 (11.8; NA)	
Negative	37 (84)	48.1 (26; 88.0)	
**Diagnosis**			0.425
Prior 2016	30 (68)	33.5 (24.8; 88.0)	
Since 2016	14 (32)	45.8 (27.3; NA)	
**Recurrence**			**0.009**
Yes	19 (43)	32.4 (24.8; 54.4)	
No	25 (57)	88.0 (33.5; 213)	
**Chemotherapy and/or immunotherapy**			
Yes	16 (36)	111.2 (13.4; 150.3)	
No	28 (64)	45.8 (24.9; 64.7)	0.325
n = 33			
**Tumor location**			0.793
Peripheral	22 (67)	48.1 (24.8; 112.6)	
Central	11 (33)	33.5 (13.4; NA)	
**Inflammatory changes**			0.823
Yes	10 (30)	41.7 (1.1; 112.6)	
No	23 (70)	48.1 (24.6; 140.4)	
**Pleura tag**			0.177
Yes	15 (45)	140.4 (17.0; NA)	
No	18 (55)	41.7 (24.6; 111.2)	
**Tumor contours**		45.8 (24.8; 55.1) 112.6 (1.1; NA)	**0.037**
Spiculated	21 (64)		
Smooth	10 (30)		
Lobulated (excluded)	2 (6)		
**Carcinoembryonic antigen** (CEA) (µg/L)	(n = 23)		
<5 µg/L	14 (61)	106.8 (32.4; 140.5)	
≥5 µg/L	9 (39)	33.5 (4.5; 111.2)	0.195
**Cytokeratin-19-fragment** (CYFRA21-1) (µg/L)	(n = 21)		
<3.3 µg/L	17 (81)	55.1 (13.4; 111.2)	
≥3.3 µg/L	4 (19)	54.4 (32.4; NA)	0.214
**Neuron-Specific Enolase**	(n = 18)		0.191
(NSE) (µg/L)			
<12.5 µg/L	14 (78)	54.4 (13.4; 111.3)	
≥12.5 µg/L	4 (22)	112.6 (55.1; NA)	

AC-ASC: Adenocarcinomapredominant adenosquamous carcinoma, BAL-ASC: structurally balanced adenosquamous carcinoma, SCC-ASC: squamous-cell carcinoma-predominant adenosquamous carcinoma, percentage may not equal to 100 due to rounding.

## Data Availability

The data presented in this study are available on reasonable request from the corresponding author. The data are not publicly available due to the valid European General Data Protection Regulations.

## Supplementary Materials

The following supporting information can be downloaded at https://www.mdpi.com/article/10.3390/jcm13195711/s1. Table S1. Tumor markers in patients with curative surgical treatment. Table S2. PD-L1 expression according to structural components.

## Abbreviations

AC	Adenocarcinoma
AC-ASC	Adenocarcinoma-predominant adenosquamous carcinoma
ALK	Anaplastic lymphoma kinase
ASC	Adenosquamous carcinoma
BAL	Structurally balanced adenosquamous carcinoma
CEA	Carcinoembryonic antigen
CYFRA21-1	Cytokeratin-19-fragment
CI	Confidence interval
EGFR	Epidermal growth factor receptor
Del19	Exon 19 deletion
HR	Hazard Ratio
Ins20	Exon 20 insertion
L858R	Exon 21 Leu858Arg substitution
NSCLC	Non-small cell lung cancer
NSE	Neuron-specific enolase
OS	Overall survival
PD-L1	Programmed death-ligand 1
PL 1-3	Invasion of the pleura visceralis
ROS-1	ROS protooncogene 1
SCC	Squamous cell carcinoma
SCC-ASC	Squamous cell carcinoma predominant adenosquamous carcinoma
TTF-1	Thyroid transcription factor 1
V1/2	Vascular invasion
WHO	World Health Organization
