# Supplementary material for: Adenosquamous Carcinoma of the Lung: Survival, Radiologic Findings, PD-L1, and Driver Mutations
Source: J Clin Med. 2024 Sep 25;13(19):5711. doi: 10.3390/jcm13195711 (PMC11476650; doi:10.3390/jcm13195711)
Supplement: Supplementary file 1 [file jcm-13-05711-s001.zip › jcm-3198206-supplementary.pdf]

Supplement material

Illini et al., Adenosquamous carcinoma of the lung: Survival, radiologic findings, PD-L1, and driver mutations

Assessment of EGFR, ROS-1, ALK mutations and PD-L1 status

For EGFR mutations, the formalin-fixed paraffin-embedded specimens were dissected and the genomic DNA was extracted and purified using the cobas® DNA Sample Preparation Kit (Roche) or the DNA FFPE Tissue Kit (Qiagen). EGFR mutations were assessed using either the cobas ® 4800 System (Roche) or the TheraScreen EGFR-Mutation Kit (Qiagen) with the LightCycler® 480 (Roche) for real-time PCR analysis. To detect ALK mutations, IHC was performed using Ventana anti-ALK (D5F3) CDx Assay (Roche). Additionally, ALK-FISH (Abbott/Vysis) was applied for determination of ALK status, if ALK-IHC results were inconclusive. For analysis of ROS-1 mutations, IHC was conducted using the antibody clones D4D6 (Cell Signaling Technology) or EP282 (Epitomics) or the Ventana ROS1 (SP384) antibody clone. To assess ROS-1 gene status in cases of inconclusive IHC results, ROS-1 Gene Fusions Detection Kit (Amoy Diagnostics) was utilized. PD-L1 expression was analyzed using antibody clone E1L3N (Cell Signaling Technology), QR1 (Diagomics), 28.8 (Dako), or the Ventana PD-L1 (SP263) Assay (Roche). The tumor proportion score (TPS) of PD-L1 was reported as the percentage of ≥100 viable tumor cells with complete or partial PD-L1 staining and classified into TPS <1%, TPS 1 to 49%, TPS 50 to 89% and ≥90%.

Tables

Supplemental Table S1. Tumor markers in patients with curative surgical treatment

**Tumor markers**	
**Carcinoembryonic antigen** (CEA) (µg/l)	(n = 23)
Median (Range)	4.4 (1-41.8)
Groups, n (%)	
< 5 µg/l	14 (61)
≥ 5 µg/l	9 (39)
**Cytokeratin-19-fragment** (CYFRA21-1) (µg/l)	(n = 21)
Median (Range)	2 (0.6-22.4)
Groups, n (%)	
< 3.3 µg/l	17 (81)
≥ 3.3 µg/l	4 (19)
**Neuron-Specific Enolase** (NSE) (µg/l)	(n = 18)
Median (Range)	10.4 (3.9-16.6)
Groups, n (%)	
< 12.5 µg/l	14 (78)
≥ 12.5 µg/l	4 (22)

Percentage may not equal to 100 due to rounding.

Supplemental Table S2. PD-L1 expression according to structural components

	**AC-ASC**<br>n = 17	**SCC-ASC**<br>n = 26	**BAL-ASC**<br>n = 1
PD-L1 status, n (%)			
Negative	8 (47)	8 (31)	0 (0)
1-49%	4 (24)	16 (62)	1 (100)
≥50%	5 (29)	2 (8)	0 (0)

AC-ASC: Adenocarcinoma predominant adenosquamous carcinoma, BAL-ASC: structurally balanced adenosquamous carcinoma, SCC-ASC: Squamous-cell carcinoma predominant adenosquamous carcinoma, Percentage may not equal to 100 due to rounding.
